# Vision-related quality of life following glaucoma filtration surgery

**DOI:** 10.1186/s12886-017-0466-7

**Published:** 2017-05-12

**Authors:** Kazuyuki Hirooka, Eri Nitta, Kaori Ukegawa, Akitaka Tsujikawa

**Affiliations:** Department of Ophthalmology, Kagawa University Faculty of Medicine, 1750-1 Ikenobe, Miki, Kagawa 761-0793 Japan

**Keywords:** Quality of life, Glaucoma filtration surgery, VFQ-25

## Abstract

**Background:**

To evaluate vision-related quality of life (VR-QOL) following glaucoma filtration surgery.

**Methods:**

A total of 103 glaucoma patients scheduled to undergo glaucoma filtration surgery. Prior to and at three months after glaucoma filtration surgery, trabeculectomy or EX-PRESS, all patients completed the 25-item National Eye Institute Visual Function Questionnaire (VFQ-25). A total of 48 patients underwent combined cataract and filtration surgery. The clinical data collected pre- and postoperatively included best-corrected visual acuity (BCVA) and intraocular pressure (IOP).

**Results:**

The IOP decreased significantly from 19.0 ± 8.1 mmHg to 9.7 ± 3.9 mmHg (*P* < 0.001). Preoperative VFQ-25 composite score (65.8 ± 15.6) was similar to the postoperative score (67.8 ± 16.6). A significantly improved VFQ-25 composite score (pre: 63.2 ± 17.1, post: 67.7 ± 17.8; *P* = 0.001) was observed in the patients who underwent combined cataract and filtration surgery. There was a significant association between the BCVA changes in the operated eye and the changes in the VFQ-25 composite score (r = −0.315, *P* = 0.003).

**Conclusions:**

Although glaucoma filtration surgery by itself did not decrease the VR-QOL in glaucoma patients, there was significant improvement in the VR-QOL after the patients underwent combined cataract and glaucoma filtration surgery.

## Background

Glaucoma is one of the principal causes of low vision and blindness globally. Glaucoma affects approximately 5% of Japanese adults aged 40 years and older [1]. Previous studies have shown that intraocular pressure (IOP) is the major risk factor for developing glaucoma, with surgical intervention required for glaucoma patients who develop visual field (VF) deterioration or exhibit progression of optic nerve damage despite receiving maximal tolerable pharmacological IOP-lowering therapy, laser therapy, or both. Among all of the incisional glaucoma procedures, trabeculectomy is the most commonly utilized surgery [2]. One other alternative that was designed and created to control the IOP is the EX-PRESS drainage device (Alcon Laboratories, Fort Worth, TX).

In 2001, the National Eye Institute developed the 25-Item Visual Function Questionnaire (VFQ-25) [3]. This vision-related quality of life (VR-QOL) instrument was designed to both assess a patients’ perception of their visual function and their QOL [3]. When using this questionnaire, it is possible to measure the visual function in 11 different domains. The specific items that are examined include general, color, driving, and peripheral vision, the presence of ocular pain, visual function for near and distance activities, and the vision-specific social functioning, mental health, role difficulties, and dependency. When trying to determine the effect that glaucoma has on the VR-QOL, the VFQ-25 is frequently used to track the outcome. Previous studies that examined the VFQ-25 reported it to be useful, sensitive, and specific when investigating relationships between the VF disturbance due to glaucoma and the QOL in glaucoma patients [4–9].

Other studies have examined patients with proliferative diabetic retinopathy, retinal detachment, macular hole, and epiretinal membrane and tried to determine the influence of vitrectomy on the VR-QOL [10–12]. The results from these studies demonstrated that vitrectomy improved the VR-QOL of these patients. A further study also suggested that cataract surgery might be able to improve the VR-QOL in the patients who undergo the procedure [13]. However, it has been noted that some patients complain of decreased visual quality after undergoing filtration surgeries, even though their visual acuity (VA) has returned to its preoperative level. Even though such findings are not all that uncommon, there have yet to be any studies that have specifically examined the changes in the VR-QOL that occur in glaucoma patients after glaucoma filtration surgery. Therefore, the purpose of this study was to evaluate the VR-QOL in patients with glaucoma following glaucoma filtration surgery.

## Methods

### Patients

We included 103 consecutive glaucoma patients who underwent filtration surgery at Kagawa University Hospital between June 2015 and March 2016. The study protocol was approved by the Institutional Review Board of the Kagawa University Faculty of Medicine. Prior to entry and participation in this research study, all participants provided both standard consent for surgery and written informed consent. After being enrolled in the study, baseline ophthalmic evaluations were performed in each of the subjects. These evaluations included IOP testing, dilated fundus examination with stereoscopic biomicroscopy of the optic nerve head using slit-lamp and indirect ophthalmoscopy, and VA testing with refraction. Patients with ocular disorders, except for mild refractive errors and mild cataract, were excluded from the study. In addition, we also excluded patients who had undergone bilateral filtration surgery within 3 months of the current study.

### Surgical procedure

All surgeries were performed by a single surgeon (KH). All eyes were prepared and draped after administering retrobulbar anesthesia with lidocaine 2%. Following the placement of a corneal traction suture using 5–0 silk suture, the surgeon dissected a fornix-based conjunctival flap, and then created a one-half thickness scleral flap (approximately 3.5 × 3.5 mm). Subsequently, mitomycin C (MMC) was applied to the sclera over the proposed scleral flap site, with 6 to 8 sponges containing 0.04% MMC solution positioned and maintained in the subconjunctival space for 3 to 5 min. After removal of the sponges, the area was copiously irrigated with 250 ml of physiologic saline. Peripheral iridectomy was performed during the trabeculectomy, with a block of clear cornea and trabecular meshwork tissue at the edge of the corneoscleral bed removed during the procedure. Using 6 to 7 monofilament 10–0 nylon sutures, the scleral flap was sutured, with the sutures adjusted to ensure that a small amount leakage would be observed around the scleral flap margin without causing any shallowing of the anterior chamber.

For the EX-PRESS procedure, an incision in the anterior chamber parallel to the iris was created using a 26-G needle. After first suturing the scleral flap using 2 to 4 monofilament 10–0 nylon sutures, the conjunctiva was then closed via the use of 10–0 nylon sutures at the edges of the incision, with one or more horizontal mattress sutures placed centrally. A balanced salt solution was used to reform the anterior chamber, with the wound checked for leaks. A sterile eye patch and shield were applied after the instillation of a corticosteroid/antibiotic ointment.

### Data collection

The logarithm of the minimum angle of resolution (logMAR) best-corrected visual acuity (BCVA) was used to bilaterally assess the corrected VA, with the logMAR BCVA values obtained before and at 3 months after the surgery. Program 30–2 of the Humphrey Field Analyzer (HFA) (Carl Zeiss Meditec, Dublin, CA) was used to collect the VF data, with only reliable VF data utilized. HFA reliability criteria (<25% fixation losses, <15% false-positive errors) were applied. When determining the test reliability, the false-negative rate was not used as an indicator [14]. Calculations of the mean deviation (MD) scores in both eyes of the subject were performed by VF testing using the HFA. All of the VF data were collected during a 3-month period prior to the surgery for all of the study subjects.

### VFQ-25

Patients completed the VFQ-25 at 1 day before and at 3 months after the surgery. The VFQ-25 consists of 12 subscales and a composite. Since general health is excluded from the calculation, the composite is defined as the average score of 11 subscales. The items assigned to the 12 subscales included general health, general, driving, color and peripheral vision, the visual function for near and distance activities, the presence of ocular pain, and the vision-specific social functioning, mental health, role difficulties, and dependency. The scoring for the subscales ranged from 0 to 100 points, with a score of 100 indicating the highest possible function or minimal subjective impairment. A Japanese version of the VFQ-25 was used in this study, with a few modifications made in order to suit the Japanese culture and lifestyle. A previous study that examined this modified VFQ-25 questionnaire for both reliability and validity has reported that it accurately measured the VR-QOL in Japanese individuals [15].

### Statistical analysis

The mean scores for each of the VFQ-25 subscales and the composite score were calculated. Pre- and postoperative results were compared using a Student’s *t*-test. A Spearman’s correlation coefficient was used to investigate the relationship between the pre- and postoperative VFQ-25 composite scores, the relationship between the preoperative scores, and the changes in the VFQ-25 composite scores. To investigate the relationship between the various explanatory variables and the VFQ-25 composite scores, we performed a multiple regression analysis of the data collected before and after the surgery. The variables that were examined included the operated eye MD, fellow eye MD, operated eye BCVA, and the fellow eye BCVA. All statistical analyses were performed using SPSS version 19.0 (IBM, New York, NY). A *P* value of less than 0.05 was considered to be statistically significant. Data are presented as the mean ± standard deviation.

## Results

Table 1 summarizes the glaucoma patient background data. Among the 103 total glaucoma patients, 71 were phakic and 32 were pseudophakic. Combined cataract surgery was performed in 48 patients while there were 78 patients who underwent trabeculectomy.

**Table 1:** Clinical Data for the study group

Age (yrs)	65.8 ± 11.6
Gender (M/F)	51/52
Diagnosis	
POAG	44
NTG	34
EG	3
SG	19
PACG	3
Lens status	
Phakic	71
Pseudophakic	32
Mean deviation (dB)	
Operated-eye	−18.2 ± 6.9
Fellow-eye	−11.27 ± 8.3
BCVA	
Operated-eye	0.169 ± 0.316
Fellow-eye	0.133 ± 0.417
Glaucoma medications	
PGA + β blocker + CAI	35
PGA + β blocker + CAI + brimonidine	43
PGA + CAI + brimonidine	5
Others	25

*M* male, *F* female, *POAG* primary open-angle glaucoma, *NTG* normal-tension glaucoma, *EG* exfoliation glaucoma, *SG* secondary glaucoma, *PACG* primary angle closure glaucoma, *IOP* intraocular pressure, *BCVA* best correlated visual acuity, *PGA* prostaglandine analogue, *CAI* carbonic anhydrate inhibitor

There was a significant decrease in the IOP at 3 months after the surgery (*P* < 0.001; Table 2). There was no significant difference noted for the BCVA when the preoperative values were compared with those at 3 months after the surgery (Table 2).

**Table 2:** Change in IOP and BCVA before and after Surgery

	Before	After	*P* value
IOP (mmHg)	19.0 ± 8.1	9.7 ± 3.9	<0.001
BCVA	0.169 ± 0.316	0.18 ± 0.299	0.67

*IOP*, intraocular pressure, *BCVA* best correlated visual acuity

Table 3 summarizes the pre- and postoperative VFQ-25 questionnaire results. The postoperative results for the general vision, ocular pain and near activities were significantly higher than those found preoperatively. Tables 4 and 5 present the pre- and postoperative results for the VFQ-25 composite scores and 12 subscales in the patients who underwent trabeculectomy (trabeculectomy alone: *n* = 39) and EX-PRESS (EX-PRESS alone: *n* = 16), respectively. Significantly higher results were found for the postoperative versus the preoperative VFQ-25 questionnaire scales for the ocular pain and near activities in the patients who underwent trabeculectomy. In the patients who underwent EX-PRESS, however, similar pre- and postoperative values were found for the VFQ-25 composite score and the 12 subscales. Tables 6 and 7 present the results of the pre- and postoperative VFQ-25 composite scores and the 12 subscales found in the patients who underwent combined cataract and glaucoma surgery or glaucoma surgery alone, respectively. A significantly higher postoperative VFQ-25 composite score was observed in the patients who underwent combined cataract and glaucoma surgery as compared to the preoperative scores. However, there were similar pre- and postoperative VFQ-25 composite scores in the patients who underwent glaucoma surgery alone. Tables 8 and 9 show the results of the pre- and postoperative VFQ-25 composite scores and the 12 subscales found in the patients who were phakic or pseudophakic, respectively. Similar pre- and postoperative values were found for the VFQ-25 composite score.

**Table 3:** The National Eye Institute Visual Function Questionnaire (VFQ-25) Composite Score and 12 Subscales in Patients with Glaucoma before and after Surgery (*n* = 103)

VFQ-25 Questionnaire Scale	Preoperatively	Postoperatively	*P* value
General health	52.7 ± 12.0	54.3 ± 13.1	0.14
General vision	56.2 ± 15.6	59.7 ± 15.7	0.02
Ocular pain	70.9 ± 21.1	76.7 ± 19.1	0.003
Near activities	63.9 ± 17.9	66.7 ± 16.1	0.045
Distance activities	65.3 ± 16.9	66.8 ± 17.7	0.32
Social functioning	75.9 ± 16.5	77.3 ± 16.0	0.31
Mental health	62.1 ± 21.3	64.8 ± 21.6	0.13
Role difficulties	73.7 ± 20.6	73.3 ± 20.9	0.81
Dependency	75.1 ± 23.4	74.7 ± 24.6	0.81
Driving	56.6 ± 27.5	56.2 ± 31.7	0.85
Color vision	79.9 ± 19.0	80.9 ± 19.6	0.58
Peripheral vision	55.6 ± 27.8	55.9 ± 24.2	0.91
Composite score	65.8 ± 15.6	67.8 ± 16.6	0.08

**Table 4:** The National Eye Institute Visual Function Questionnaire (VFQ-25) Composite Score and 12 Subscales in Patients with Glaucoma before and after Trabeculectomy (*n* = 78)

VFQ-25 Questionnaire Scale	Preoperatively	Postoperatively	*P* value
General health	52.3 ± 11.7	54.2 ± 13.2	0.10
General vision	57.3 ± 15.7	60.7 ± 15.9	0.07
Ocular pain	71.5 ± 21.4	77.3 ± 19.2	0.01
Near activities	65.1 ± 18.0	68.9 ± 16.1	0.03
Distance activities	66.8 ± 16.9	68.3 ± 17.8	0.35
Social functioning	76.6 ± 17.0	78.6 ± 15.7	0.17
Mental health	64.5 ± 21.4	66.4 ± 22.3	0.33
Role difficulties	75.9 ± 20.6	76.4 ± 20.6	0.81
Dependency	77.0 ± 22.7	78.8 ± 22.2	0.33
Driving	59.5 ± 25.3	60.4 ± 29.4	0.66
Color vision	80.8 ± 17.5	82.1 ± 18.9	0.48
Peripheral vision	56.7 ± 21.9	58.3 ± 23.0	0.51
Composite score	67.3 ± 16.0	70.0 ± 16.8	0.07

**Table 5:** The National Eye Institute Visual Function Questionnaire (VFQ-25) Composite Score and 12 Subscales in Patients with Glaucoma before and after EX-PRESS (*n* = 25)

VFQ-25 Questionnaire Scale	Preoperatively	Postoperatively	*P* value
General health	53.9 ± 13.2	54.5 ± 13.1	0.81
General vision	52.5 ± 15.1	56.5 ± 14.6	0.14
Ocular pain	69.2 ± 20.4	74.7 ± 18.9	0.12
Near activities	59.9 ± 17.3	60.1 ± 14.6	0.94
Distance activities	60.9 ± 16.5	62.1 ± 16.8	0.70
Social functioning	74.0 ± 14.9	73.0 ± 16.3	0.73
Mental health	54.8 ± 19.6	59.9 ± 19.0	0.24
Role difficulties	66.6 ± 19.4	63.6 ± 19.4	0.46
Dependency	69.3 ± 25.1	62.0 ± 27.6	0.19
Driving	46.5 ± 32.9	41.4 ± 35.9	0.35
Color vision	77.1 ± 23.2	77.1 ± 22.0	>0.99
Peripheral vision	54.3 ± 23.4	50.0 ± 25.0	0.33
Composite score	61.1 ± 13.5	61.7 ± 14.7	0.77

**Table 6:** The National Eye Institute Visual Function Questionnaire (VFQ-25) Composite Score and 12 Subscales in Patients with Glaucoma before and after Combined Cataract and Glaucoma Surgery (*n* = 48)

VFQ-25 Questionnaire Scale	Preoperatively	Postoperatively	*P* value
General health	54.6 ± 11.8	55.3 ± 13.0	0.67
General vision	52.9 ± 15.8	58.3 ± 14.4	0.02
Ocular pain	69.7 ± 21.1	77.3 ± 20.3	0.005
Near activities	61.6 ± 20.0	66.9 ± 16.4	0.01
Distance activities	62.4 ± 18.0	66.9 ± 18.5	0.02
Social functioning	73.4 ± 18.2	77.0 ± 16.2	0.02
Mental health	60.0 ± 22.4	64.4 ± 24.3	0.047
Role difficulties	70.0 ± 23.0	71.2 ± 23.5	0.58
Dependency	71.2 ± 25.6	74.8 ± 24.7	0.12
Driving	52.7 ± 27.5	57.2 ± 31.6	0.06
Color vision	78.8 ± 18.2	81.0 ± 17.6	0.32
Peripheral vision	52.7 ± 22.3	54.3 ± 26.2	0.64
Composite score	63.2 ± 17.1	67.7 ± 17.8	0.001

**Table 7:** The National Eye Institute Visual Function Questionnaire (VFQ-25) Composite Score and 12 Subscales in Patients with Glaucoma before and after Glaucoma Surgery Alone (*n* = 55)

VFQ-25 Questionnaire Scale	Preoperatively	Postoperatively	*P* value
General health	51.1 ± 12.0	53.3 ± 13.2	0.10
General vision	59.0 ± 15.1	60.9 ± 16.7	0.39
Ocular pain	72.0 ± 21.2	76.1 ± 18.1	0.13
Near activities	65.8 ± 15.7	66.6 ± 16.0	0.70
Distance activities	68.0 ± 15.7	66.7 ± 17.1	0.54
Social functioning	78.2 ± 15.9	77.4 ± 15.9	0.72
Mental health	64.0 ± 20.4	65.1 ± 19.3	0.66
Role difficulties	76.9 ± 18.0	75.1 ± 18.4	0.48
Dependency	78.5 ± 21.0	74.5 ± 24.7	0.19
Driving	61.6 ± 25.9	56.7 ± 31.4	0.14
Color vision	81.8 ± 18.3	82.3 ± 18.4	0.87
Peripheral vision	58.2 ± 23.1	57.3 ± 22.4	0.73
Composite score	68.1 ± 13.9	67.9 ± 15.7	0.91

**Table 8:** The National Eye Institute Visual Function Questionnaire (VFQ-25) Composite Score and 12 Subscales in patients with phakic eyes before and after Glaucoma Surgery Alone (*n* = 23)

VFQ-25 Questionnaire Scale	Preoperatively	Postoperatively	*P* value
General health	55.3 ± 10.7	54.3 ± 13.0	0.56
General vision	65.1 ± 13.1	65.8 ± 13.8	0.81
Ocular pain	78.3 ± 17.3	78.6 ± 19.6	0.96
Near activities	72.2 ± 11.2	72.5 ± 12.6	0.92
Distance activities	74.0 ± 12.7	72.5 ± 15.7	0.65
Social functioning	84.8 ± 11.2	85.3 ± 13.1	0.84
Mental health	70.0 ± 16.7	72.6 ± 15.6	0.33
Role difficulties	85.3 ± 18.3	82.7 ± 16.1	0.42
Dependency	87.0 ± 16.7	85.0 ± 18.1	0.38
Driving	69.9 ± 20.1	62.5 ± 31.0	0.18
Color vision	89.1 ± 12.7	90.2 ± 12.5	0.75
Peripheral vision	69.6 ± 18.4	66.3 ± 19.4	0.42
Composite score	75.0 ± 9.7	74.4 ± 12.9	0.79

**Table 9:** The National Eye Institute Visual Function Questionnaire (VFQ-25) Composite Score and 12 Subscales in patients with pseudiphakic eyes before and after Glaucoma Surgery Alone (*n* = 32)

VFQ-25 Questionnaire Scale	Preoperatively	Postoperatively	*P* value
General health	47.6 ± 12.1	51.9 ± 13.5	0.03
General vision	53.4 ± 14.4	57.2 ± 17.0	0.13
Ocular pain	67.3 ± 22.0	74.3 ± 17.7	0.03
Near activities	60.9 ± 16.5	61.6 ± 17.3	0.78
Distance activities	62.6 ± 16.2	61.5 ± 17.0	0.71
Social functioning	72.2 ± 14.5	70.4 ± 14.9	0.55
Mental health	58.6 ± 21.6	60.1 ± 20.1	0.71
Role difficulties	70.1 ± 15.2	69.0 ± 18.5	0.79
Dependency	71.5 ± 22.0	65.7 ± 26.5	0.27
Driving	47.0 ± 32.1	45.2 ± 35.0	0.61
Color vision	75.0 ± 19.7	75.8 ± 20.2	0.85
Peripheral vision	48.3 ± 21.7	50.0 ± 22.7	0.65
Composite score	62.4 ± 13.9	62.6 ± 16.2	0.95

**Fig. 1:**
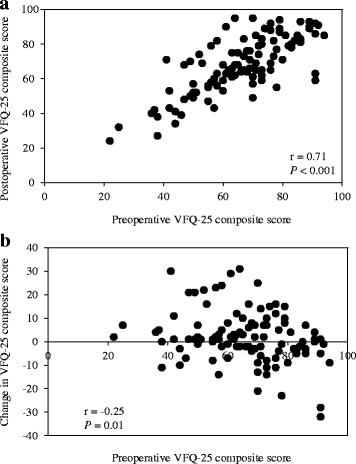
**a** Preoperative VFQ-25 composite score versus postoperative VFQ-25 composite score. **b** Preoperative VFQ-25 composite score versus changes in VFQ-25 composite score

There was a significantly correlation noted between the preoperative VFQ-25 composite scores and both the postoperative VFQ-25 composite score (r = 0.71, *P* < 0.001, Fig. 1a) and the changes in the VFQ-25 composite score (r = −0.25, *P* = 0.01, Fig. 1b).

The results of the multiple regression analyses that were performed to examine the relationships between the VFQ-25 composite score and the various explanatory variables are presented in Tables 10 and 11. As seen in Table 10, a significant correlation was observed between the preoperative fellow eye MD, the fellow eye BCVA, or the operated eye BCVA and the postoperative VFQ-25 composite score. With or without cataract surgery was found to be significantly associated with changes in the VFQ-25 composite score (Table 11).

**Table 10:** Results of Multiple Regression Analysis of Postoperative Composite score and Explanatory Variable

MD		BCVA		With or without cataract surgery
Operated eye	Fellow eye	Operated eye	Fellow eye	
−0.081	0.288*	−0.200*	−0.226*	0.048

*MD*, mean deviation; *BCVA*, best correlated visual acuity

*Significant at *P* < 0.05

**Table 11:** Results of Multiple Regression Analysis on Changes in VFQ-25 Composite score and Explanatory Variable

MD		BCVA		With or without cataract surgery
Operated eye	Fellow eye	Operated eye	Fellow eye	
−0.004	0.137	−0.099	0.096	0.227*

*MD* mean deviation, *BCVA*, best correlated visual acuity

*Significant at *P* < 0.05

## Discussion

Changes in the VR-QOL that occur after glaucoma surgery are important and therefore, need to be carefully evaluated. Although improvements in the VR-QOL may be observed in patients after they are able to decrease the number of required eye drops, the VFQ-25 does not sufficiently examine the inconvenience and cost for patients with glaucoma. Other investigators have previously reported that the QOL scores did not differ between medically and surgically treated groups [16, 17]. In the current study, we assessed the pre- and postoperative VR-QOL in glaucoma patients through the use of the VFQ-25 questionnaire.

Patients who undergo filtration surgeries sometimes complain of decreased visual quality even though their VAs have returned to preoperative levels. After this phenomenon was examined in other studies, it was suggested that changes in the corneal surface contour, anterior chamber depth, and axial length, which are factors related to refraction, had been induced by the surgical procedure [18–21]. Although other studies that examined filtration surgery patients during the initial three months after the surgery found that the VA returned to baseline during this period [22, 23], it is possible that these types of changes could have an effect on the VR-QOL following glaucoma filtration surgery. Therefore, our current study was designed to examine the potential changes in patients at three months after their surgeries through the use of a self-administered VFQ-25 questionnaire.

The results of our multiple regression analysis demonstrated that there was a significant correlation between the changes in the VFQ-25 composite score and the changes in the operated eye BCVA. Other investigations that examined VR-QOL outcomes after vitrectomy for various vitreoretinal disorders have reported finding only a weak or no correlation between the increases in the QOL and the improvement in the VA [11, 12, 24]. In contrast, Hiratsuka et al. [13] examined patients after cataract surgery and reported finding a significant correlation between the change in the VA in the eye and the improved QOL. In our current study, we found that there was a significant association between the pre- and postoperative VR-QOL as well as between the preoperative VR-QOL and the changes in the VR-QOL. Although there was a significant increase in the postoperative BCVA from 0.2239 ± 0.336 to 0.151 ± 0.256 (n = 48; *P* = 0.026) in the patients who underwent combined glaucoma and cataract surgery, we also found a significant decrease in the postoperative BCVA from 0.109 ± 0.286 to 0.206 ± 0.332 (n = 55; *P* = 0.001) in patients who underwent glaucoma surgery alone. Our current study also showed there was a significant improvement in the postoperative VR-QOL in patients who underwent combined glaucoma and cataract surgery. Thus, the increased VR-QOL that was observed after the combined cataract and glaucoma surgery was due to the improvement in the VA.

Our study also revealed that there was a significantly higher postoperative versus preoperative VFQ-25 questionnaire scale score for the ocular pain. In order to achieve target IOPs, it is common for glaucoma patients to use several different IOP lowering medications. Unfortunately, the eye drops commonly used for glaucoma therapy contain preservatives that may induce ocular surface diseases. In such cases, patients may exhibit a variety of signs that include pain or discomfort during instillation, stinging or burning or eyelid itching, foreign body sensation, and superficial punctate keratopathy [25, 26]. In contrast, patients do not need eye drops for glaucoma therapy after glaucoma surgery, provided the filtering function is working well. This may be the reason for the noted differences in the pre- and postoperative ocular surface conditions. Moreover, this difference might have also had an influence on the VFQ-25 questionnaire scale score for the ocular pain. As seen in Table 1, many glaucoma medications were used before surgery. After surgery, however, only corticosteroid and antibiotic eye drops were used. Patients who underwent EX-PRESS surgery did not exhibit any increase in their ocular pain score. This may be related to the small sample size (*n* = 25) of the current study.

The Early Manifest Glaucoma Trial previously reported that many patients with VF loss of less than 50% (e.g., VF index 50% or MD −18 dB) in the better eye rated their VR-QOL similarly to patients with no VF loss in the better eye [27]. Although the MD in the operated eye was−18.2 ± 6.9 dB, the MD in the fellow-eye was−11.27 ± 8.3 dB in the current study. VF loss in the fellow-eye, which was probably the better eye, was less than 50% in our patients. Glaucoma surgery was a predictor of poor QOL only in patients with early stages of the disease and did not influence the QOL in the more advanced patients [28]. Thus, our results support the findings of the previously published reports.

There were some limitations for our current study. First, evaluations of the VR-QOL took place at three months after the surgery. As glaucoma is a chronic progressive disease and cannot be cured by this type of surgery, patient evaluations that only use the VFQ-25 might not be able to completely reveal the effect of the glaucoma filtration surgery. In fact, the visual impairment that has been found to occur after trabeculectomy has been reported to be due to the development of cataracts [29]. Therefore, when the VR-QOL is used to evaluate patients after an extended period of time has passed since the surgery, the possibility of an effect caused by cataract progression or glaucoma progression cannot be ignored. Thus, since the VA has been reported to return to baseline during the first 3 months after filtration surgery [22, 23], we decided to investigate the VFQ-25 values at 3 months after surgery. The second potential limitation of our study is that the patients answered the questionnaire both pre- and postoperatively. This opens up the possibility that since the patients might have had a desire to please their surgeon or justify the inconvenience after the surgery, they might have tended to answer the questionnaire more positively after the surgery. However, as the VFQ-25 questionnaires were filled out anonymously, we believe that most of the patients probably answered honestly. Another limitation of our study is that we included different types of glaucoma, which can have a different prognosis and thus, probably have different effects on the VR-QOL.

## Conclusions

In conclusion, glaucoma filtration surgery by itself did not decrease the VR-QOL in the glaucoma patients examined in this study. Furthermore, there was a significant improvement in the VR-QOL of the glaucoma patients after they underwent combined cataract and glaucoma filtration surgery.

## Abbreviations

BCVA: Best-corrected visual acuity
HFA: Humphrey Field Analyzer
IOP: Intraocular pressure
logMAR: Logarithm of the minimum angle of resolution equivalents
MD: Mean deviation
MMC: Mitomycin C
VA: Visual acuity
VF: Visual field
VFQ-25: 25-Item Visual Function Questionnaire
VR-QOL: Vision-related quality of life
